# Use of the integrated health interview series: trends in medical provider utilization (1972-2008)

**DOI:** 10.1186/1742-5573-9-2

**Published:** 2012-03-30

**Authors:** Mike Davern, Lynn A Blewett, Brian Lee, Michel Boudreaux, Miriam L King

**Affiliations:** 1 National Opinion Research Center, University of Chicago, 1155 East 60th Street, Chicago, IL 60637, USA; 2 School of Public Health, University of Minnesota, State Health Access Data Assistance Center (SHADAC), 2221 University Ave, Suite 345, Minneapolis, MN 55414, USA; 3 Minnesota Population Center, Room 50 Willey Hall, 7931, 225 19th Ave S, Minneapolis, MN 55455, USA; 4 State Health Access Data Assistance Center (SHADAC), 2221 University Ave, Suite 345, Minneapolis, MN 55414, USA

**Keywords:** Health care utilization, Cohort study, NHIS, Medicare

## Abstract

The Integrated Health Interview Series (IHIS) is a public data repository that harmonizes four decades of the National Health Interview Survey (NHIS). The NHIS is the premier source of information on the health of the U.S. population. Since 1957 the survey has collected information on health behaviors, health conditions, and health care access. The long running time series of the NHIS is a powerful tool for health research. However, efforts to fully utilize its time span are obstructed by difficult documentation, unstable variable and coding definitions, and non-ignorable sample re-designs. To overcome these hurdles the IHIS, a freely available and web-accessible resource, provides harmonized NHIS data from 1969-2010. This paper describes the challenges of working with the NHIS and how the IHIS reduces such burdens. To demonstrate one potential use of the IHIS we examine utilization patterns in the U.S. from 1972-2008.

## Background

The National Health Interview Survey (NHIS) is an annual cross-sectional household survey of the civilian, non-institutionalized U.S. population sponsored by the National Center for Health Statistics. Since 1957 the NHIS has collected detailed information on health conditions, health status, health behaviors, healthcare utilization, and insurance coverage. These rich data covering the past half century can illuminate the effect of policy changes, reveal long-term trends and differentials in health status and care, and allow analysts to study relatively rare conditions or small population subgroups by pooling data from multiple years. Yet despite the temporal depth of this rich data resource, multi-year analysis of the NHIS has been limited to a few researchers who have had the time and temerity to tackle linking the annual survey data across years. Studies such as Cohen et al.'s 48 year study of health insurance coverage [1], Chay and colleagues' investigation of hospital utilization, mortality, and the introduction of Medicare [2], or Reither et al.'s 26 year cohort study of obesity [3] were once only possible by NCHS staff or by study teams with sophisticated data analysis resources. Obstacles to long-term analyses included an overwhelming number of source files and a massive volume of documentation; periodic changes in the survey design; and perennial changes in the survey questions, variable names, and coding schemes.

These obstacles to multi-year analysis have recently been overcome by the Integrated Health Interview Series (IHIS). The IHIS provides free online access to thousands of consistently-coded and fully-documented NHIS variables from 1969 to the present (at http://www.ihis.us). The project involved implementing an Internet-based data access system that provides public health information on over five million respondents. IHIS multiplies the value of NHIS data by allowing researchers to make consistent comparisons throughout four decades of dramatic change in public health, and thus to study the health status of Americans as a dynamic process. The uses of these data are many. However, in coming years, researchers and policy makers will have a special need for rich health data, such as the IHIS, to fully understand the effects of the Affordable Care Act-the most dramatic change to the American health system since the introduction of Medicare and Medicaid.

In this work, we describe the IHIS and its underlying foundation, the National Health Interview Survey. This description updates readers to IHIS developments since its introduction by Johnson et al. in 2008 [4]. Among other improvements, the IHIS now includes a web-based analysis tool that allows users to access and analyze decades of data directly from their web-browser. We also provide an example of one potential use of the IHIS through an analysis of prior year medical utilization between 1972 and 2008.

### National health interview survey

The NHIS is the leading source of information on the health of the U.S. population. Depending on funding levels, the annual survey has covered between 60,000 and 130,000 persons per year, with a high (approximately 90%) household response rate. Data are collected in face-to-face interviews administered by the National Center for Health Statistics (NCHS) in cooperation with the U.S. Census Bureau [5-9]. The NHIS has been in continuous operation since 1957, making it the longest-running and most extensive health survey in the world.

The NHIS is comprised of a core survey and supplemental surveys. The core survey provides data on demographic characteristics, socioeconomic status, general health, disability, injuries, and access to and use of health care for all family members [10]. Since 1997, additional questions covering specific health conditions and other topics (e.g., HIV testing) have regularly been asked of one randomly-selected adult and child per family. Supplemental surveys varying from year to year have covered a host of special topics, such as complementary and alternative medicine and cancer control and epidemiology [6,11,12]. For the years 1986 to 2004 the NHIS can be linked to the National Death Index, allowing researchers to examine predictors of mortality as well as covariates of illness and disability. NHIS data have been a valuable resource for monitoring the nation's health, used by policy makers and scholars alike. The rich array of data from the NHIS have been indispensable for studying diverse health-related topics, including: how health status, access to medical care, insurance coverage, morbidity, and morbidity/mortality differ according to social class, race, ethnicity, and living arrangements [13-24]; the relationship between disability and various social and economic outcomes such as labor force participation, poverty status, and receipt of disability benefits [25-29]; public education and outreach [30,31]; and alcohol and tobacco consumption [32-34]. NHIS is also used more than any other data source for tracking progress toward the quantifiable public health goals articulated in the Healthy People 2020 initiative. This broad-based collaborative effort among Federal, State, private, and nonprofit organizations sets national disease prevention and health promotion objectives to be achieved by the end of the decade, to improve the quality and years of healthy life, and to eliminate health disparities.

### Advantages of IHIS

Despite the temporal and topical scope of NHIS data, there have been surprisingly few analyses of the survey's key public health and health policy variables over time. By contrast, multi-year analyses using IHIS are the norm rather than the exception. Over 70 percent of the 4,361 data extracts from our 1,592 registered users included data from more than 1 year, and over 40 percent included data from 10 or more years.

Explaining the difference in how the NHIS and IHIS are used lies in the contrasting paths that researchers must follow to work with multiple NHIS public use files versus IHIS data. In the first (NHIS) case, a researcher who wishes to study change over time must search for relevant variables scattered across more than 500 data files and reams of accompanying documentation; s/he must cope with ubiquitous changes in variable names, codes, question wording and universes (particularly for the period prior to 1997); and s/he must merge files and consult separate codebooks for each survey section and sample year. The task is so daunting, time-consuming, and prone to unintentional errors that most researchers have opted to focus on data from a single year or, at most, a handful of years.

The IHIS was specifically designed to overcome these barriers and to facilitate chronological analysis. The methods used to create the IHIS database are modeled after the Integrated Public Use Microdata Series, a harmonized resporitory of U.S. Census data from 1850-2000 [35]. A detailed description of IHIS methods is available in Johnson et al. [4]; however, a brief methodological note is worth mention here. The IHIS team harmonizes NHIS variables across time using "translation tables" - a tool that is used to create a common variable from disparate codes across time. Variables are created using a composite coding scheme in which the first digit applies to all years, the second digit to a broad subset, and subsequent digits to fewer years. In this manner the NHIS data can be made compatible across time with no information loss.

IHIS users can locate variables relevant to a particular research topic by consulting a user-friendly website that displays variable availability across years at a glance, by viewing variables grouped by topic (e.g., tobacco use, asthma), and/or by using an online search tool. Variable-specific online documentation provides information about meaning, universes, appropriate weights, comparability issues, NHIS source variables, question wording, and year-specific codes and frequencies. While a common survey question like "Have you smoked 100 cigarettes in your life?" may appear in differently-named variables in the original NHIS files (in the case of this example, under 11 different names across 30 years), IHIS variables are given consistent names and coded consistently across time, without loss of information. Using the online data extraction system, IHIS users merge files on the fly to create a data extract with just the years and variables they need. Moreover, users can easily link IHIS data to original NHIS variables that have not yet been added to the IHIS system. Comparing the IHIS to the original NHIS is facilitated by an online concordance tool that maps IHIS variables to their NHIS counterparts. In addition to its online documentation system, IHIS provides user workshops and has a dedicated user support team (for upcoming workshops see http://www.ihis.us/ihis/coming_events.shtml).

As noted earlier, there are many reasons why multi-year analysis of data collected through the NHIS is desirable. While NHIS sample size is large (e.g., almost 90,000 persons in 2009), much of the information is collected for only one sample adult and/or one sample child per family. For researchers who wish to study relatively rare health conditions and/or relatively small population subgroups, pooling data across years is the only feasible approach. To address the effect of policy changes and long-term trends in health behaviors, health conditions, and health care access and use, analysis across multiple decades may be required.

To facilitate data pooling and trend analysis, IHIS consulted with NCHS statisticians to modify the survey design variables (STRATA and PSU) so that they can be used when examining data from one year or from many years. By identifying common patterns across the original weights and sampling schemes, the staff reduced the hundreds of file-specific weights to seven named weights (with guidance about the appropriate weight to use included in each variable description). Episode-level condition data from the period prior to 1997 are being converted into dichotomous variables largely comparable to the individual-level condition variables for 1997 forward. This innovation will open the way to studying long-term trends, differentials, and correlates of such major health problems as diabetes, asthma, hypertension, and stroke, from the 1960s to the present. IHIS will soon be adding "family pointer variables" that can be used in conjunction with the data access system to attach the characteristics of a co-resident mother, father, and/or spouse or partner to an individual's record. And while the number of consistently-coded variables available through IHIS now exceeds 7,000, that number will approximately double in the next 3 years, to encompass nearly all NHIS variables from the 1960s forward.

To foster the use of the IHIS for those without access to appropriate statistical computing resources, the IHIS can now be analyzed online. This service is facilitated by UC-Berkeley's Computer Assisted Survey Methods Program (http://sda.berkeley.edu/). Users can generate cross-tabulations, compare means, and conduct linear regression, logistic, and probit regression from their web-browser. All analyses are conducted with appropriate attention to the survey design and results are presented in tables and graphs.

### An applied example of the IHIS

As a concrete example of the power of IHIS to unlock the full potential of the NHIS, we examine a healthcare utilization measure to highlight changes in provider utilization over time. This indicator, time since last provider visit (*the IHIS variable DVINT*), is available for 37 years, from 1972 to 2008. While long-term changes and cohort differences in health care utilization may be interesting in their own right, they could be employed in a public policy context. For example, with the coverage expansions included in the Affordable Care Act (ACA), there is specific interest in assessing any changes in access or physician utilization in a reformed health care environment. Indeed a key goal of the ACA is to increase utilization in groups that currently have poor access. However, increased demand facilitated by coverage expansion may lead to provider shortages, an unintended consequence of the policy. Assessing such outcomes is only possible with readily accessible and rich baseline data such as the IHIS. Moreover, a complete understanding of the ACA's utilization effect can only be arrived at with a solid grasp of the temporal patterns that have shaped utilization behavior in the past. Such patterns include the long term impact of past policies (knowledge of which will help guide contemporary research questions) and the impact of age, birth cohort, and secular time trends. Such research is only possible with a data source such as the IHIS.

To shed light on these issues we conducted a cohort study of provider utilization from 1972-2008. Our goal was not to resolve unsettled scientific questions, but to demonstrate the power of the IHIS in illuminating policy-relevant issues. All data and documentation was obtained from the IHIS website.

The outcome of interest was based on a healthcare utilization variable collected by asking the respondent to approximate the interval since the respondent last saw or talked to a medical provider (DVINT). From 1972 to 1981 the question was worded "about how long has it been since you saw or talked to a medical doctor?" Beginning with the 1982 NHIS survey, the question changed slightly to include "medical assistants" along with doctors. This implied that professionals such as a nurse practitioner or a physician assistant would also be counted. Beginning in 1997, the question wording changed again to "medical doctor or other healthcare professional." Additionally, beginning in 1997 the question universe changed such that this question was only asked of a sample adult and a sample child within each household. In order to control for the changes in the construction of the survey item and survey operations in the NHIS over time, we included an indicator variable for "survey periods" (1972-1981, 1982-1996, and 1997-2005) in the multivariate analysis to control for measurement changes.

When answering the question the respondent was instructed to choose from several categorical responses ranging from "never" to "more than 5 years ago." For this analysis we created a dichotomous variable indicating if the respondent had visited a medical provider within the past 12 months. Responses that were "unknown" or refusals (approximately 1.2 percent of the total sample) were coded as missing.

We constructed six 10-year birth cohorts based on the reported age (variable name AGE) of the respondent, using the 1972 survey as the starting point. With each successive survey year, the age range for each cohort shifted by one year. For example, cohort #1 in the 1972 survey included respondents age 13-22 (born between 1950-1959), in the 1973 survey this included respondents age 14-23. The birth cohorts are: 1950-1959, 1940-1949, 1930-1939, 1920-1929, 1910-1919, and 1900-1909. For data privacy protection, the NHIS top-coded their age variable at 85 for several of the survey years. Therefore we no longer followed a cohort once the upper age range reached an average of 85. For example, cohort 6 (born 1900-1909) has the upper range of 85 for the 1985 survey. The six cohorts were modeled as separate indicator variables. Estimates were not calculated for respondents born outside these cohort years.

Age was classified in three-year groups (18-20, 21-24 and so forth, to 81-84) along with a category for age less than 18 and a category for age 85 and older, modeled as separate indicator variables to observe any age effects in utilization. Changes in utilization over successive survey years were examined by creating a centered survey year variable where the mean survey year -1989, in our analysis - represents zero and remaining years were assigned a value relative to 1989. Modeling this variable in a continuous fashion allowed for observing utilization changes while also minimizing collinearity with age and cohort variables. We also created indicator variables for "survey periods" (1972-1981, 1982-1996, and 1997-2005) to determine if utilization patterns changed as a result of questionnaire alterations.

Several demographic covariates were defined as follows. Sex (SEX) was modeled as an indicator variable. Race (RACEA) was modeled with three indicator variables (White, African-American, and Other Race). Employment (EMPSTAT) was defined as employed, employed but not at work, unemployed, and not in labor force. Marital status (MARSTAT) was classified as married, widowed, divorced, separated, and never married. Education (EDUC) status was defined as less than high school, a high school diploma, some college, college degree, and post-Bachelors. Self-reported health status (HEALTH) was measured on a five-point scale from 1982-2008 and a four-point scale from 1972-1981 and in both cases the scale was dichotomized into "poor" (fair and poor) and "at least good health" (excellent, very good, and good).

### Analytic methods

The health status variable was introduced during the 1972 survey, consequently we restricted the analyses to data from 1972-2008. Furthermore, since the question universe for several demographic covariates changed over 37 years, we limited our estimations to respondents aged 18 and older, as all items used in the regressions have been measured on those over 17 years of age over this time period. For example the marital status and employment status universes have changed during the period we examined the NHIS. From 1963-1981 the marital status universe was over 17 and in 1982 it changed to over 13 years of age. The employment status universe changed from over 16 years of age from 1963-1981 to over 17 years of age from 1982 to the present. By focusing our analysis on only those 18 and over we always have observations for these variables that we enter in the logistic regression analysis as control variables.

First, we estimated the mean age and proportion with medical provider visits within the past year for each cohort over the 37 years of data. These estimates were then plotted to visualize trends in utilization over time by cohort. Because the issue of disentangling cohort effects, time effects, and age effects is an important concern [36] we also run a series of logistic regression models. These models all use the utilization variable we constructed to incrementally examine age effects, cohort and time effects. We also use the models to enter in several other control variables. The control variables used in the logistic regressions include: sex, race, employment, marital status, education, and survey period. Model 1 has the control variables and age. Model (2) adds birth cohort to the age variable and controls. Model (3) adds a centered year variable to Model 2.^a^. We then used the result of Model 3 and marginal effects to create figures to better understand the impact of specific variables in our model while holding others constant at their mean.

The analysis was conducted using Stata statistical software (SE version 9.2) to account for the complex survey design of the NHIS. The person weight (PERWEIGHT) variable was used for all observations as we restricted our variables to those collected on all adults 17 years of age or older (and were not part of the sample adult interview where the more restrictive weights are needed). We used Taylor series linearization for variance estimation [37], and followed the current IHIS recommendation to use the method Korn and Graubard [38] refer to as "situation 2" (page 280), the concatenated design period pooling approach for pooling data from one survey over multiple years and sample designs. The IHIS data make this easy by making the PSU variable and the strata variable consistent within design periods but unique between them. When pooling multiple years worth of data, researchers can identify the cluster or PSU as the variable "PSU" and the strata and the IHIS variable "STRATA" for 1985 and beyond. Lowess smoothing techniques were used with all graphs presented [39].

## Results

**Figure 1:**
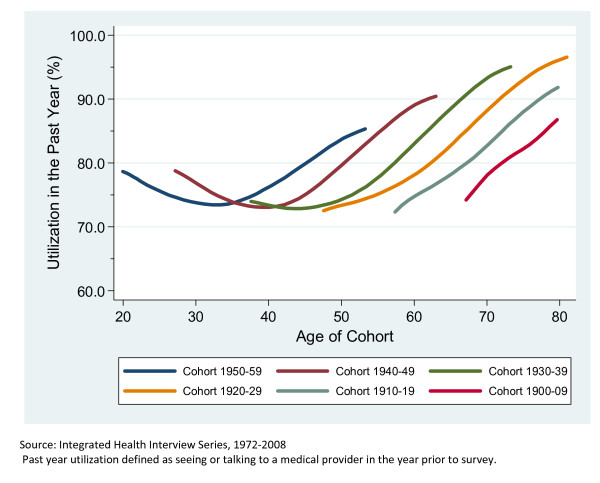
**Rate of Past Year Utilization by Cohort Age**. Adults in the United States, 1972-2008.

We first present simple unadjusted changes in utilization by birth cohort and age in Table 1 and Figure 1. Table 1 contains the utilization results for the six birth cohorts at four specific age categories. At 48 years of age, the utilization ranges from 70.9% to 83.6% having seen a medical provider in the past year. The first two cohorts (1920-1929 and 1930-1939) are not significantly different from each other at age 48, but both are different from successive cohorts. The 1940-1949 cohort utilized at 76.4% and the 1950-1959 cohort utilized at 83.6%. A similar pattern emerges with the other age ranges as well. At 58 years of age the 1910-1919 cohort utilized at 73.0% and the 1940-1949 cohort utilized at a significantly higher rate of 88.9%. At 68 years of age the 1900-1909 cohort utilized at a rate of 75.4% and the 1930-1939 cohort utilized at a significantly higher rate of 93.5%. The final data point shows that the 1900-1909 cohort utilized at a rate of 84.2% when they were 78 years old and the 1920-1929 cohort utilized at a significantly higher rate of 95.2%.

**Table 1:** Rate of Past Year Utilization by Age and Birth Cohort, Adults in the United States, 1972-2008

	Average Age of Birth Cohort			
	
	48 Years	58 Years	68 Years	78 Years
Birth Cohort 1900-1909	--	--	75.4%	84.2%

Birth Cohort 1910-1919	--	73.0%	80.3%	89.6%

Birth Cohort 1920-1929	70.9%	74.4%	86.1%	95.2%

Birth Cohort 1930-1939	71.2%	79.6%	93.5%	--

Birth Cohort 1940-1949	76.4%	88.9%	--	--

Birth Cohort 1950-1959	83.6%	--	--	--

Source: Integrated Health Interview Series, 1972-2008Past year utilization defined as seeing or talking to a medical provider in the year prior to survey

Both Table 1 and Figure 1 show initial strong signs of a cohort effect in that each successive cohort is utilizing medical providers more frequently than the last at the same age and that the divergence seems to be growing over time. Figure 1 also shows that utilization for each cohort begins to level off suggesting a ceiling effect with the rates. These results are further investigated through analysis that adjusts for changes in the NHIS survey instrument and changes in self-reported health status over time.

Table 2 shows a series of regression models. Model 1 includes age and the various control variables of health status, age group, birth cohort, sex, education level, employment, marital status, race, and NHIS survey design period. In Model 2 we introduce birth cohort into the model and the birth cohorts show the strong association that is clear in Table 1 and Figure 1, but does not greatly impact the effect of the age. However, in Model 3 when we introduce a variable that controls for the passage of time into the regression model (representing global changes in the incidence of medical care utilization from one year to the next), the birth cohort effects are greatly diminished (although not entirely eliminated). We use this model to demonstrate the relative importance of the time variable in a series of adjusted figures similar to Figure 1, but based on marginal effect estimates of changes over time.

**Table 2:** Logistic Regressions of Past Year Utilization.

		*MODEL 1*		*MODEL 2*		*MODEL 3*
	**OOR^a^**	**(95% CI^b^)**	**OR**	**(95% CI)**	**OR**	**(95% CI)**

***Health Status***						

Excellent, Very Good or Good	-	-	-	-	-	-

Fair or Poor	2.95	(2.90-3.01)	2.97	(2.92-3.02)	2.97	(2.91-3.02)

***Age Group***						

18-20	-	-	-	-	-	-

21-24	0.89	(0.87-0.92)	0.91	(0.88-0.93)	0.88	(0.85-0.91)

25-28	0.71	(0.69-0.73)	0.75	(0.73-0.78)	0.71	(0.68-0.73)

29-32	0.61	(0.60 - 0.63)	0.67	(0.64-0.69)	0.60	(0.58-0.62)

33-36	0.55	(0.54-0.57)	0.61	(0.59-0.64)	0.53	(0.51-0.55)

37-40	0.52	(0.50-0.53)	0.59	(0.56-0.61)	0.48	(0.46-0.50)

41-44	0.50	(0.48-0.52)	0.59	(0.56-0.61)	0.47	(0.45-0.49)

45-48	0.52	(0.51-0.54)	0.64	(0.61-0.66)	0.49	(0.46-0.51)

49-52	0.57	(0.55-0.59)	0.71	(0.68-0.74)	0.52	(0.50-0.55)

53-56	0.60	(0.58-0.62)	0.78	(0.74-0.82)	0.55	(0.52-0.59)

57-60	0.66	(0.64-0.69)	0.91	(0.86-0.96)	0.62	(0.58-0.66)

61-64	0.71	(0.69-0.74)	1.05	(0.99-1.11)	0.69	(0.64-0.74)

65-68	0.83	(0.80-0.87)	1.36	(1.28-1.45)	0.85	(0.79-0.92)

69-72	1.01	(0.97-1.05)	1.82	(1.70-1.94)	1.10	(1.01-1.19)

73-76	1.25	(1.19-1.30)	2.41	(2.24-2.59)	1.40	(1.28-1.54)

77-80	1.40	(1.32-1.48)	2.85	(2.62-3.09)	1.61	(1.46-1.78)

81-84	1.73	(1.58-1.89)	3.61	(3.23-4.03)	2.01	(1.78-2.27)

85-99	2.46	(1.81-3.33)	4.56	(3.33-6.23)	2.47	(1.80-3.39)

***Birth Cohort***						

1950-1959			-	-	-	-

1940-1949			1.01	(1.00-1.03)	1.11	(1.09-1.14)

1930-1939			0.93	(0.91-0.96)	1.14	(1.11-1.18)

1920-1929			0.80	(0.77-0.83)	1.08	(1.03-1.13)

1910-1919			0.60	(0.58-0.63)	0.89	(0.84-0.95)

1900-1909			0.41	(0.38-0.43)	0.66	(0.61-0.71)

***Centered Year***						

Centered Year					1.02	(1.01-1.02)

Centered Year Squared						

Adults in the United States, 1972-2008

Source: Integrated Health Interview Series, 1972-2008.Past year utilization defined as seeing or talking to a medical provider in the year prior to survey

^a ^Odds Ratio

^b ^95% Confidence Interval

Note: * indicates p < .01

Note: All models adjusted for sex, race, employment, marital status, education, and survey period

**Figure 2:**
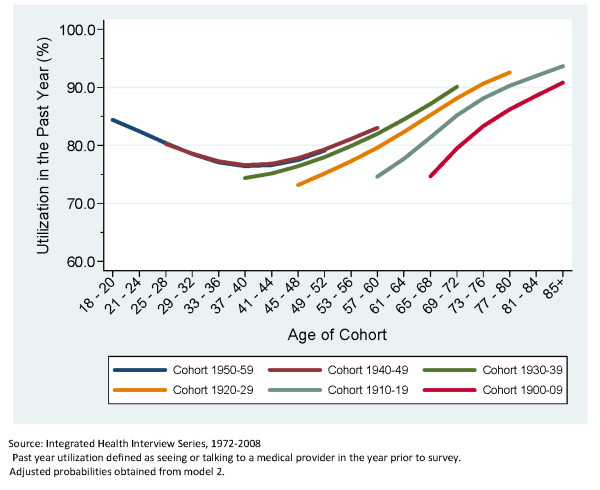
**Predicted Probability of Past Year Utilization, Adjusted for Age, Birth Cohort and Selected Demographics**. Adults in the United States, 1972-2008.

**Figure 3:**
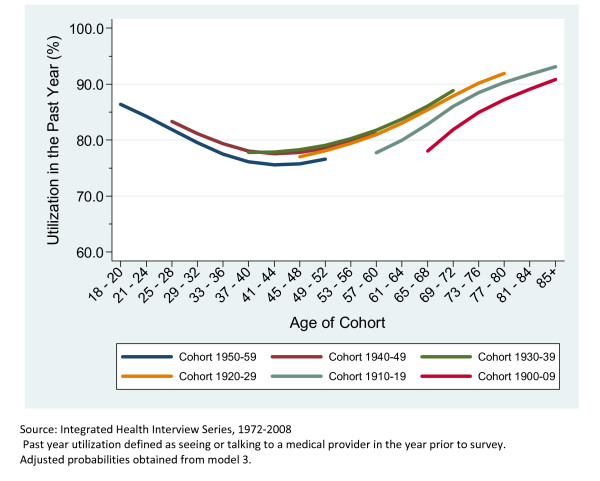
**Predicted Probability of Past Year Utilization, Adjusted for Age, Birth Cohort, Survey Year, and Selected Demographics**. Adults in the United States, 1972-2008.

**Figure 4:**
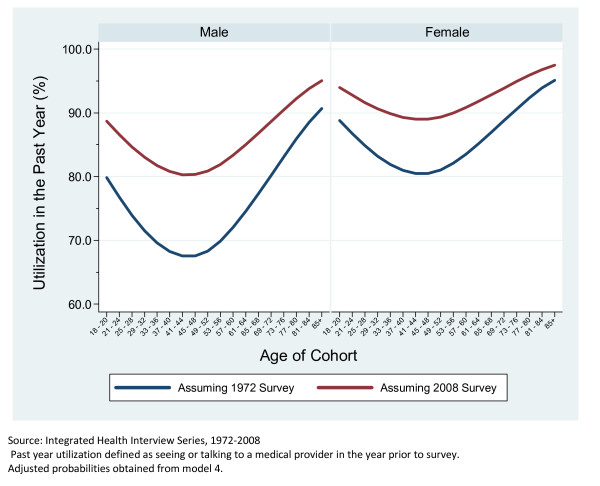
**Predicted Probability of Past Year Utilization Evaluated at Survey Years 1972 and 2008, Adjusted for Age, Birth Cohort, Survey Year, and Selected Demographics**. Adults in the United States, 1972-2008.

Figure 2, which is based off of Model 2, shows a greatly diminished cohort effect after controlling for age, health status, education, race, marital status and employment. Figure 3 is based off of Model 4 and it is clear that after adjusting for the impact of a global increase in the incidence of medical care utilization for each additional year of data there is far less impact of birth cohort on utilization. However, there is still a clear difference between the earliest birth cohort and those that come after. Figure 4 clearly shows the large cumulative impact of the passage of time on the incidence of medical care utilization for both men and women. Figure 4 presents the marginal effects estimates of time at the two extremes (if everyone were observed in the first year versus everyone was observed in the final year).

## Discussion

The IHIS is a unique national data resource that provides health services researchers, epidemiologists, and social scientists ready access to 40 plus years of data on health behaviors, health conditions, utilization, and access to care. Through the NHIS, the United States has the most historically extensive set of survey based health data in the world. However, the chronological breadth of this resource often remains idle due to the complex task of linking yearly files into a consistent time series. The IHIS provides a free and easy-to-use portal to the NHIS, with the hope that long-run analyses of health and health care in the United States will become the norm rather than the exception.

There are several limitations on our analysis that must be noted. When conducting research on surveys that have been conducted annually for over 37 years, there are inevitably many changes that occur both in the operations of fielding the survey but also in the methods used to collect that data. We have tried to control for some of these changes through the harmonization process, but clearly we cannot control for them all. It's possible that some change over time could be influencing our findings. Also, we would like to note that we are using repeated cross-sections and not following the actual same people over time so that there could be issues in the composition of these populations that is confounding our analysis. Finally, we would like to have a more comprehensive measure of health care utilization. While we acknowledge that our measure of utilization is crude, we believe it is this same simplicity that makes it a unique indicator of change and an appropriate, available measure that can used fairly consistently.

In this paper we demonstrated the power of the IHIS in conducting a cohort analysis in an effort to disentangle age and period effects on one measure of provider utilization over the time period from 1972-2008. We found a robust relationship between time, measured by an ordinal centered-year variable, and having at least one medical provider contact per year in the United States.

Our findings are consistent with other studies that document an increase in physician visits over shorter time periods and more targeted groups of people. The Center for Health System Change found "the proportion of Medicare seniors seeing a doctor at least once in the previous year increased to 92% in 2003, up from 87% in 1997. Likewise, Medicare seniors reported having 5.5 physician visits a year in 2003 on average, up from 5.2 visits in 1997" [40].

Others have utilized cohort analysis to address age, period and time effects but its applications in studying health care are largely underutilized. Cohort analysis of health care use has been a modeling tool used by social scientists for many years. One of the earlier works by Wolinsky et al. [41] used elderly age cohorts from the 1972, 1976 and 1980 National Health Interview Surveys to demonstrate significant age and period effects on physician and hospital utilization. Further analysis found significant substitution of hospital-based services for ambulatory care and the level of family social supports to explain increase in utilization by age over time.

MaCurdy and Geppert [42] use a similar "cohort-time empirical framework" to identify high-cost Medicare users using 10 years of Medicare claims data for a 5 percent sample of all Medicare beneficiaries. They found expenditure patterns rising similarly across different age cohorts with the top 5-10 percent with the highest share of expenditures. In addition, the researchers were also able to identify higher growth rates for younger Medicare enrollees compared to older Medicare enrollees, yet the aggregate year affects were large and consistently positive. Jonathan Skinner, in his comment on the paper, concluded that at least for this cohort of young and old elderly, annual aggregate "shock" -namely changes in Medicare payment and provider practice patterns - influences future spending [42].

Researchers in the Netherlands [43] use cohort analysis to disentangle age, period, and cohort effects of health status of the elderly using self-reports of functional limitations and data from the Longitudinal Aging Study Amsterdam that follows 3,107 individuals aged 55-85 during the period 1992-1999. They find increasing age and period effects but no cohort effects in their analysis. That is, the "prevalence of functional limitations at older ages grew during the 90s in the Netherlands" and this finding was explained by adverse period effects-restrictions on health care in the Netherlands - after controlling for demographic and socioeconomic status. They conclude with the importance of including period effects in modeling functional or health status and the elderly.

More recently, researchers at the Federal Reserve Bank of Minneapolis used data from the Sweden Human Mortality Database from 1861 to 2005 on ages 64 and younger to examine period and cohort effects on mortality [44]. They found continuous improvements over time in mortality at all ages with larger improvements for young adult ages and smaller improvement for later age cohorts. The findings illuminate the impact of influences of cumulative impact cohort trajectories and highlights the role of cumulative cohort theory in studying human mortality in a social context.

## Conclusion

The ease of accessibility of the IHIS harmonized data series will facilitate the application of this methodology as multiple years of data become more readily available. In our basic analysis, we demonstrate the value of looking at age cohorts and health care utilization. While we cannot assess the underlying causes of what makes last year's 72-year-olds more likely to utilize healthcare than today's 72-year-olds, our results do provide evidence of a significant change in our utilization measure over time. What is critical is to acknowledge the ever-changing complexity of society over time. Describing the health care utilization of today's elderly may tell us very little about that of the future elderly if such analysis is based on research that does take into account the different cohorts of the population who will be aging.

## Competing interests

The authors declare that they have no competing interests.

## Authors' contributions

MD developed the conceptual framework and methodological approach and helped to write the method and discussion sections. LB wrote the primary draft of the manuscript and facilitated several revisions. BL conducted the analysis and wrote the initial methods section. MK and MB assisted with developing and writing the introduction. All authors have read and approved the final manuscript.

## Endnote

^a ^Centering of year simply makes the year have a 0 mean (in other words subtracts 1990 from the year) to deal with the collinearity issues from putting in age, cohort and year all into the same model. We also tested an additional model which had a centered year squared variable to test for a non-linear relationship but it did not alter the results so is not included but is available upon request form the corresponding author.
